# Related neural networks underlie suppression of emotion, memory, motor processes as identified by data-driven analysis

**DOI:** 10.1186/s12868-023-00812-5

**Published:** 2023-08-24

**Authors:** Karisa J. Hunt, Lindsay K. Knight, Brendan E. Depue

**Affiliations:** 1 Department of Psychological and Brain Sciences, University of Louisville, 2301 S, 3rd St., Louisville, KY 40292 USA; 2 Present Address: Insightec Ltd., Chicago, IL USA

**Keywords:** Inhibitory control, Emotion regulation, Memory suppression, Motor inhibition, Independent component analysis, Intrinsic connectivity networks

## Abstract

**Background:**

Goal-directed behavior benefits from self-regulation of cognitive and affective processes, such as emotional reactivity, memory retrieval, and prepotent motor response. Dysfunction in self-regulation is a common characteristic of many psychiatric disorders, such as PTSD and ADHD. This study sought to determine whether common intrinsic connectivity networks (ICNs; e.g. default mode network) are involved in the regulation of emotion, motor, and memory processes, and if a data-driven approach using independent component analysis (ICA) would successfully identify such ICNs that contribute to inhibitory regulation.

**Methods:**

Eighteen participants underwent neuroimaging while completing an emotion regulation (ER) task, a memory suppression (Think/No-Think; TNT) task, and a motor inhibition (Stop Signal; SS) task. ICA (CONN; MATLAB) was conducted on the neuroimaging data from each task and corresponding components were selected across tasks based on interrelated patterns of activation. Subsequently, ICNs were correlated with behavioral performance variables from each task.

**Results:**

ICA indicated a common medial prefrontal network, striatal network, and frontoparietal executive control network, as well as downregulation in task-specific ROIs.

**Conclusions:**

These results illustrate that common ICNs were exhibited across three distinct inhibitory regulation tasks, as successfully identified through a data-driven approach (ICA).

## Introduction

Few goals can be accomplished in the absence of inhibitory control, as the ability to suppress diversions to purposive behavior is paramount to the completion of almost any task. Inhibitory control is a facet of executive function composed of many processes, such as the regulation of emotional reactivity, the suppression of memory retrieval, and the inhibition of prepotent motor response. The importance of these realms of self-regulation is particularly evident in their absence—dysfunction in these areas is a primary diagnostic criterion for many psychiatric disorders, such as post-traumatic stress disorder (PTSD), obsessive-compulsive disorder (OCD), and attention deficit hyperactivity disorder (ADHD) [1]. Though inhibitory control is ubiquitous, the exact neural mechanisms and interactions underlying its various domains remain unknown.

Many notable studies have unveiled the primary neural regions underlying the inhibition of emotional reactivity [18], memory retrieval [10], and motor response [6], as well as shared variance across psychological domains in neural regions purported to down-regulate additional regions responsible for behavioral output [19]. Frameworks exist in which ‘control’ neural regions putatively mediate the regulation of activation in ‘effector’ regions of interest (ROIs)—such as the amygdala (AMY) for emotion regulation, hippocampus (HPC) for memory suppression, and primary motor cortex (PMC) for motor inhibition [19]. For example, it has been hypothesized that the striatum controls (and can entirely stop) the updating of the flow of information from subcortical regions to the cortex, allowing ongoing congruence between actions and the goals of emotion regulation, memory suppression, and motor inhibition [25]. Furthermore, the lateral prefrontal cortex (lPFC) has been shown to play a fundamental role in the downregulation of activation and communication between sensory cortex (i.e. vision, audition), with additional support from the medial prefrontal cortex (mPFC) downregulating regions commonly associated with affect (e.g. AMY, insula [INS]) [17, 19, 42]. However, to our knowledge, no study has examined these interactions using a data-driven approach to uncover common neural networks.

In the context of functional magnetic resonance imaging (fMRI), independent component analysis (ICA) is a stable, model-free, data-driven method for exploring functional connectivity (simultaneous activation within a network of functionally activated brain regions). These areas of simultaneous activation are referred to as intrinsic connectivity networks (ICNs) and exhibit remarkable stability across time and analysis [29]. The independent components that ICA separates multidimensional signals into are maximally non-Gaussian, and the analysis does not depend on prior assumptions about the data or use underlying neural processes to explain it. This allows for the translatable identification of distinct neural networks and their functional interactions without assuming specific cognitive processes associated with them [14].

Well-known ICNs include the dorsal attention network (DAN), which aids in top-down attentional orientation to volitional action, the ventral attention network (VAN), which aids in bottom-up attentional reorientation to salient stimuli, and the frontoparietal control network, involved in many facets of inhibitory control (such as directed attention, task switching, goal-directed action, and self-monitoring), as it facilitates interaction between other networks and allows for flexible task updating [15, 44]. Though precursory models have painted a picture of response inhibition as occurring in distinct and discrete areas of cortex (e.g. the role of the right inferior frontal cortex [IFC] in motor response inhibition; Congdon et al. [16]), more recent evidence indicates response inhibition is better described as “one example of a broader class of control processes that are supported by the same set of frontoparietal … domain-general networks [that] exert control by modulating local lateral inhibition processes, which occur ubiquitously throughout the cortex” [26].

In this study, we sought to discover whether ICA could reliably detect changes in functional connectivity in such potentially common underlying executive and cognitive control ICNs as a result of an increase in task-based behavioral inhibition. Data-driven analysis of ICNs across-task is a largely underexplored method, with other procedures such as network analysis and graph theory historically emerging in the forefront. Specifically, no studies to date have examined ICNs across different domains of inhibitory control. Many studies using the aforementioned methods implicate the right middle frontal gyri (MFG) and the orbitofrontal cortex (OFC) in downregulating amygdalar response to regulate emotional reactivity [21, 37, 38, 42]. Other studies implicate the right MFG in controlling the HPC to regulate memory retrieval [3, 10, 12, 18, 22, 36]. Further studies associate the right MFG, anterior cingulate cortex (ACC), or pre-supplementary motor area (pre-SMA) with downregulating output to the motor cortex, leading to the subsequent inhibition of prepotent motor response [6, 7, 16, 24].

In light of these prior findings, we hypothesized that the control networks potentially engaged in the following tasks would enable the subsequent downregulation of task-specific effector areas, facilitating inhibitory control over prepotent responses. During an emotion regulation task, we expected to find a medial prefrontal ICN and a likely right-lateralized executive control ICN, leading to subsequent deactivation in the task’s specific effector area, the amygdala. During a memory suppression task, we expected to find a likely right-lateralized executive control ICN, leading to subsequent deactivation in the task’s specific effector area, the hippocampus. During a motor inhibition task, we expected to find a striatal ICN and a likely right-lateralized executive control ICN, leading to subsequent deactivation in the task’s specific effector area, the primary motor cortex. We predicted the presence of right-lateralized networks as activation in the right hemisphere is often associated with stimulus withdrawal or avoidance, whereas activation in the left hemisphere is often associated with stimulus approach or arousal [23, 39, 50]. Overall, we expected to observe increases in positive functional connectivity in the aforementioned areas across three tasks eliciting distinct domains of inhibitory control.

## Materials and methods

### Participants

Eighteen healthy adults were included in the final analyses for this study (10 females, 8 males; *M* age = 21.5, *SD* = 2.3). All participants were self-reported right-handed native English speakers with normal or corrected-to-normal vision and hearing and disclosed no history of psychiatric or neurological disorders. These 18 participants were a resampling of two independent studies, in order to utilize robust behavioral data. Seven participants from an initial 25 were excluded for inadequate behavioral task performance (no suppression rate in the emotion regulation and memory suppression tasks combined) or incomplete neuroimaging data. Participants with inadequate behavioral task performance were excluded case-wise to obtain robust and consistent ICNs. Participants were recruited through an online University of Colorado Boulder-based website and were compensated for their participation. Written informed consent was obtained from each participant before any experimental sessions and all experimental protocols followed were approved by the University of Colorado Boulder’s Institutional Review Board prior to the beginning of data collection.

### Procedure

During a single session at the University of Colorado Boulder, participants were briefed on MRI protocol and task instructions and asked to complete three tasks measuring the three inhibitory control domains of interest. The Emotion Regulation (ER; [37, 38]) task assessed top-down emotional reactivity regulation. The Think/No-Think (TNT; [18]) task examined memory retrieval suppression. The Stop Signal (SS; [13]) task evaluated prepotent motor response inhibition. The timing of the session was as follows: TNT scans (24 min), high-resolution structural scans (8 min), SS scans (10 min), diffusion tensor imaging (DTI) scans (12 min), ER scans (12 min), and resting-state scans (6 min); *Total time: 72 min*. High-resolution structural, DTI, and resting-state scans were conducted between tasks to allow for appropriate participant rest. As the present study aimed to investigate individual differences in a within-subject repeated-measures design while incorporating a task with high emotional valence, all participants were administered tasks in the order *TNT–SS–ER* to prevent any across-task influence from heightened emotional responses.

### Task design

#### Emotion regulation task

During the ER task, participants were shown a series of pictures with high negative emotional valence (e.g. car accidents, houses on fire) from the International Affective Picture System (IAPS; [30]), and instructed either to actively feel or to inhibit (suppress) their emotions related to the images. During the Suppress condition (indicated by a red border surrounding the presented image), participants were instructed to “Passively view the picture and remove yourself from any attached feeling.” During the Feel condition (indicated by a green border), participants were instructed to “View the picture and focus on the emotion it conveys.” After viewing each image, participants rated the intensity of their emotional reactions using a seven-point Likert scale (1 = not at all intense, 7 = extremely intense). Each participant rated 24 previously viewed images (Feel trials *N* = 12, Suppress trials *N* = 12) and 12 additional novel images.

The task followed a blocked design with 60-second rest periods featuring fixation trials at the end of each block. To establish a low-level baseline, additional fMRI baseline blocks were presented at the beginning of each trial block, in which participants exclusively viewed neutral images. These baseline blocks included four repetitions of four novel neutral images, each shown for four seconds. In each condition, 12 distinct IAPS images with high negative emotional valence (counterbalanced) were used, matched for both valence and arousal. Each image appeared in four different blocks, totaling 48 s per condition. Each trial consisted of a four-second image presentation followed by a two-second inter-trial interval.

#### Think/no-think task

Prior to the TNT task, participants underwent training to memorize 24 face-picture (cue-target) pairs of IAPS images with neutral emotional valence. Each pair was displayed for four seconds. Participants were then presented with only a cue (neutral face) and asked to select which of two neutral target images were originally paired with the cue. Both target options were previously shown throughout the training phase to eliminate novelty effects. Training continued until participants achieved 97.5% accuracy (23 out of 24 pairs). During the experiment, participants were presented with a cue for 3.5 s (followed by a 500-ms intertrial interval) and instructed to either recall the associated target image (Think condition) or attempt to prevent its retrieval (No-Think condition). The No-Think condition was indicated by a red border around the presented image, the Think condition by a green border, and the Baseline condition (in which eight novel faces were shown) by a yellow border.

During the Think condition, participants were instructed to “Think of the picture previously associated with the face.” During the No-Think condition, participants were told “Do not let the previously associated picture come into consciousness.” During the Baseline condition, participants were instructed to “Passively view the face.” Lastly, a cued recall test was administered to assess retrieval performance. During the cued recall test, participants were presented with each cue and asked to provide a description of the target association they had previously learned. Within each condition (Think, No-Think, Baseline), participants viewed the cues 12 times. Presentation order was pseudorandomized using in-house scripts. Jittered fixation trials were interspersed in a pseudorandom order, with a variable timing of 500–4000 ms. The eight cues not presented in the experimental phase served as a zero-repetition behavioral baseline, allowing assessment of normal levels of recollection accuracy.

#### Stop signal task

During the SS task, participants were presented with either a ‘stop’ or ‘go’ signal and instructed to press a button only when the ‘go’ signal appeared, refraining from pressing it before the ‘go’ signal or at the ‘stop’ signal. These signals were presented as basic shapes, as delineated by the experimental design of Chatham et al. ([13]). Participants completed 240 two-choice reaction time trials, composed of either an arrow pointing right or an arrow pointing left. In 25% of trials, these arrows were obscured by a stop signal (a white box) following a variable delay (Go trials *N* = 180, Stop trials *N* = 60). Participants were instructed to inhibit their prepotent motor response (not press any buttons) when the stop signal was present. The stop signal delay was adjusted using an adaptive algorithm based on the participant’s performance (Fig. 2). The initial delay was set at 100 ms, decreasing by 50 ms for each unsuccessful trial and increasing by 50 ms for each successful trial. Each trial was followed by a two-second inter-trial interval. To establish a low-level baseline, jittered fixation trials consisting of three circles were included. These fixation trials were interspersed in a pseudorandom order with variable timings ranging from 500 to 4000 ms. Pseudorandom design ordering was implemented using in-house scripts.

**Fig. 1:**
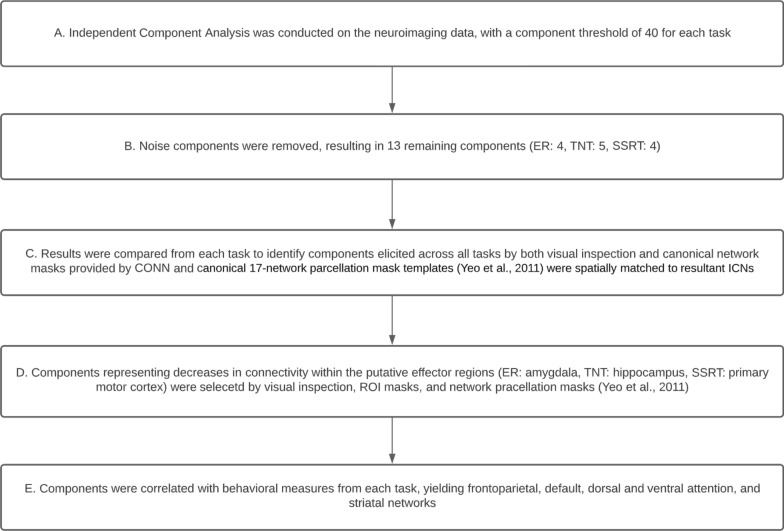
Flow of analyses

### Behavioral performance indices

The inhibition task data provided behavioral performance indices for later regression analyses. In the ER task, participants rated the intensity of their emotional reactions to the 24 images (Feel trial *N* = 12, Suppress trial *N* = 12) viewed, as well as additional novel images (Novel trial *N* = 12), using a seven-point Likert scale (1 = not at all intense, 7 = extremely intense). These ratings were later analyzed to compare Feel and Suppress trial types with the Baseline trial type. This comparison was made to assess whether participants’ volitional employment of inhibitory control influenced their rating of emotional reaction intensity to the stimuli. Additionally, inhibition indices were calculated as follows: *Inhibition Index = Baseline rating – Suppress rating*.

In the TNT task, inhibition indices were calculated as follows: *Inhibition Index = % accuracy, Baseline performance – % accuracy, No-Think performance.* Think accuracy and Baseline accuracy were defined as correct item recall, while No-Think accuracy was defined as appropriate item forgetting. The accuracy percentages of Think trial type and No-Think trial type were compared with the accuracy percentage of the Baseline trial type to evaluate the behavioral impact of participants attempting to employ volitional inhibitory control over their reactions to the presented stimuli.

Stop signal reaction time (SSRT) was computed as follows: where n = individual’s error rate on SS trials. For the purpose of ICA network evaluation, both inaccurate and accurate responses to stop and go signals were considered simultaneously at a between-conditions contrast of *[0.5, 0.5]*as a participant’s *attempted accuracy*.

*For additional methodological information, see Depue et al. [19].

## Neuroimaging methods

### Imaging data acquisition

Structural MRI images were acquired using a Siemens 3-T MAGNETOM Trio MR scanner at the University of Colorado Boulder. A 12-channel head coil was used for radiofrequency reception. Participants were given headphones to receive instructions and foam padding was added to minimize head motion within the coil. Structural images were obtained via a *T*_1_-weighted magnetization-prepared rapid gradient-echo sequence (MPRAGE) in 192 sagittal slices. Structural imaging parameters were as follows: echo time (TE) = 1.64 ms, repetition time (TR) = 2350 ms, flip angle = 7.0°, field of view (FoV) = 256 mm, and voxel size = 1.0 mm^3^. Scan parameters were consistent for all imaging sessions. Functional blood oxygenation level-dependent (BOLD) images were acquired using gradient-echo *T*_2_*-weighted echoplanar imaging. Functional imaging parameters were as follows: TE = 25 ms, TR = 2000 ms, flip angle = 67°, FoV = 240 mm, voxel size = 3.4 mm^3^ in 31 axial slices, using a 64 × 64 matrix, with a 1-mm slice gap. Slices were oriented obliquely along the anterior commissure – posterior commissure (AC–PC) line. The first four volumes from each subsequent run were discarded to allow for magnetic field equilibration.

## Imaging analysis

### Functional analysis

Functional connectivity analyses were conducted using the CONN toolbox, version 18.b based on SPM12 in the 2019a version of MATLAB. CONN’s default functional and anatomical preprocessing pipeline was applied, which included: functional realignment and unwarping, slice-timing correction, outlier identification (using 99% liberal setting), co-registration, direct segmentation and normalization, and functional smoothing (at a 6-mm full-width at half-maximum [FWHM]). Following preprocessing, CONN’s default denoising pipeline was applied, which combined two general steps: linear regression of potentially confounding effects in the BOLD signal using Ordinary Least Squares (OLS), and temporal band-pass filtering.

Potentially confounding effects were accounted for through the implementation of an anatomical component-based noise correction procedure (aCompCor), which included noise components from cerebral white matter (CWM; five components) and cerebrospinal fluid (CSF) areas, estimated participant-motion parameters, and identified outlier scans or scrubbing. The resulting residual BOLD timeseries were then band-pass filtered at 0.008 - inf. Hz, as these parameters benefit from retaining higher-frequency information fitting event-related tasks. Stimuli onsets and durations were specified in the CONN toolbox, allowing the BOLD timeseries to be divided into task-specific blocks based on the experimental design. Block regressors were convolved with the canonical hemodynamic response function (HRF), resulting in weighted connectivity metrics viewable either by condition or by contrast.

### Functional connectivity

ROI-to-ROI metrics were computed to characterize connectivity between all pairs of ROIs in a predefined set of regions. ROI-to-ROI connectivity (RRC) matrices represent the level of functional connectivity between pairs of ROIs. Each element of an RRC matrix is defined as the Fisher-transformed (variance stabilizing) bivariate correlation coefficient between a pair of ROI BOLD timeseries. Weighted seed-based connectivity (wSBC) maps were generated to characterize condition-specific functional connectivity strength. wSBC maps were computed using a weighted least squares (WLS) linear model with temporal weights identifying each experimental condition (i.e. condition-specific boxcar timeseries convolved with the canonical HRF). A standard second-level general linear model (GLM) analysis of functional connectivity matrices was applied to produce a single statistical matrix of *T-* or *F-* values, characterizing the effect of interest (i.e. differences in connectivity between two conditions) among all possible pairs of ROIs. False discovery rate (FDR) corrected *p*-values were computed using the standard Benjamini and Hochberg [9] algorithm.

To identify ICNs involved in emotion, memory, and motor regulation, a component threshold of 40 (default) was set for each task (Fig. 1a). Noise and motion components were identified and removed by CONN’s artifact removal toolbox (ARTtoolbox), resulting in 40 total components, consistent with the selected component threshold (Fig. 1b). Results were examined within individual tasks to identify a common ‘general’ set of elicited components via visual inspection, as well as with canonical network masks provided by CONN (Fig. 1c). Additionally, we applied spatial transformation of the resultant components with regard to a canonical 17-network parcellation mask template [48] to assess the robustness of the acquired components and minimize reverse inference.

**Fig. 2:**
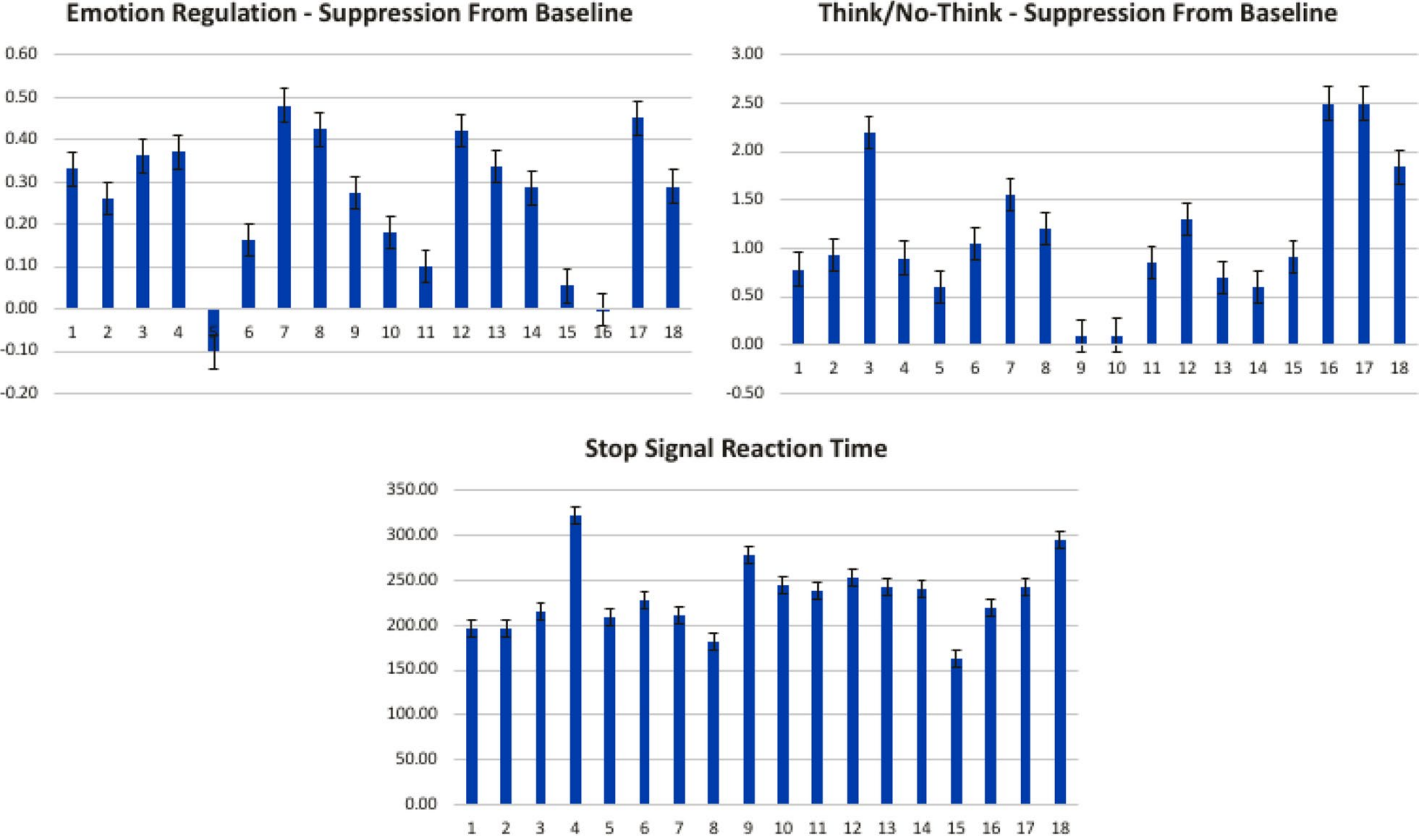
Behavioral results from ER, TNT, and SS tasks. X-axis represents participant #, Y-axis represents change in scores from baseline (ER task: beta estimate indicating the decrease in subjective rating negativity, TNT task: beta estimate of the number of items successfully suppressed, *SS task* stop signal reaction time)

Subsequently, components of interest were selected representing effector regions negatively correlated with the timeseries of the ‘general’ task-elicited components for each regulatory task (ER: AMY, TNT: HPC, SS: PMC) via the same criteria as above (Fig. 1d). The ‘general’ and effector components were regressed with behavioral suppression indices from the three tasks (*ER: Baseline rating – Suppress [negative subjective] rating, TNT: Baseline performance – % recall accuracy during No-Think condition, SSRT: attempted accurate stop signal reaction time*) to identify commonalities in brain-behavior relationships (Fig. 1e).

## Results

### Behavioral results

Behavioral performance indices are depicted in Fig. 2. These results are consistent with previous self-regulation studies [2, 6, 37]), and demonstrate that participants effectively suppressed individual levels of negative emotion, as evidenced by a reduction from baseline in subjective intensity ratings (*M* = 46.72% decrease on a linear scale of 1–4), [*t*(16) = 3.44, *p* = 0.003]. In addition, these results demonstrate that participants successfully suppressed individual retrieval of unwanted memories, as evidenced by a reduction from baseline in memory recall (*M* = 12% decrease in recalled items), [*t*(16) = 3.45, *p* = 0.003]. These results reveal a typical range of across-participant stop signal reaction time (*M* = 232.09 ms, accuracy = 50.05%). The behavioral measures of the ER and TNT tasks exhibit a significant correlation (*r* = 0.52, *p* = 0.025), whereas neither correlated with the SS task (ER: *r* = 0.04, TNT: *r* = 0.01).

## Neuroimaging results

**Fig. 3:**
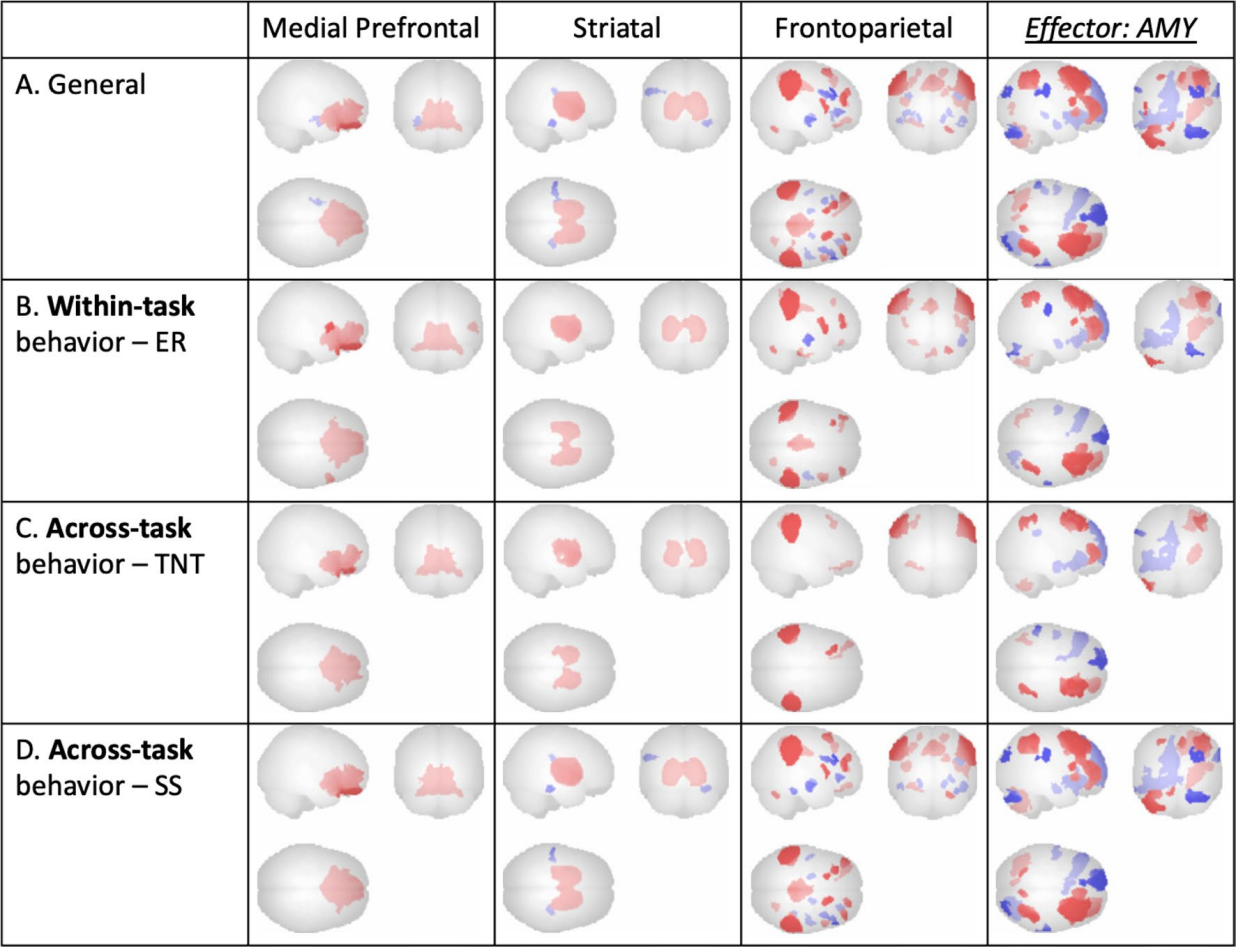
ER suppression condition ICNs: Across-task irrespective of behavioral measures (‘General’; **A**) and regressed with within-task (**B**) and across-task (**C**, **D**) behavioral measures, all at voxel threshold p < 0.001, cluster threshold p < 0.05, cluster-size p-FDR corrected, |T(17)| = 3.97 (Gaussian Random Field theory; Worsley et al. [47])

ICA results were examined within individual tasks using visual inspection and predefined network masks provided by CONN to identify component patterns across different tasks. A total of 13 patterns were identified (ER: 4, TNT: 5, SSRT: 4), including brain networks related to executive attention, the striatum, and the mPFC. These findings are illustrated in Figs. 3a and 5a, and 7a.

**Fig. 4:**
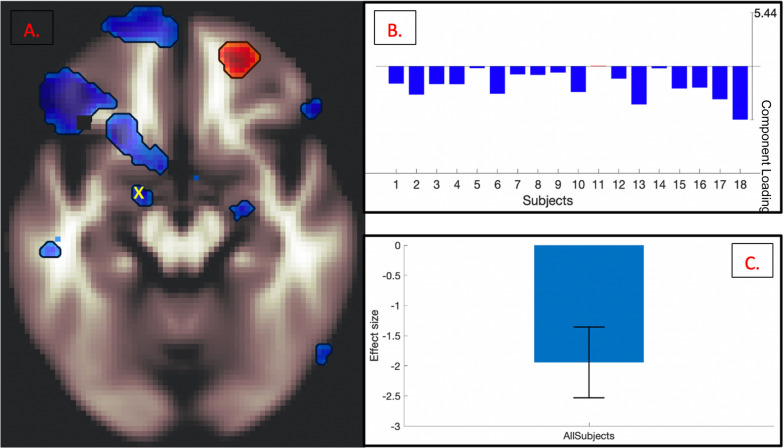
Mean across-participant peak (**A**; marked with a yellow x) of the ER task-specific effector ROI (AMY), plotted for individual participant negative component loading weights (**B**), and for effect size of all participants (**C**), p = 0.000023

**Fig. 5:**
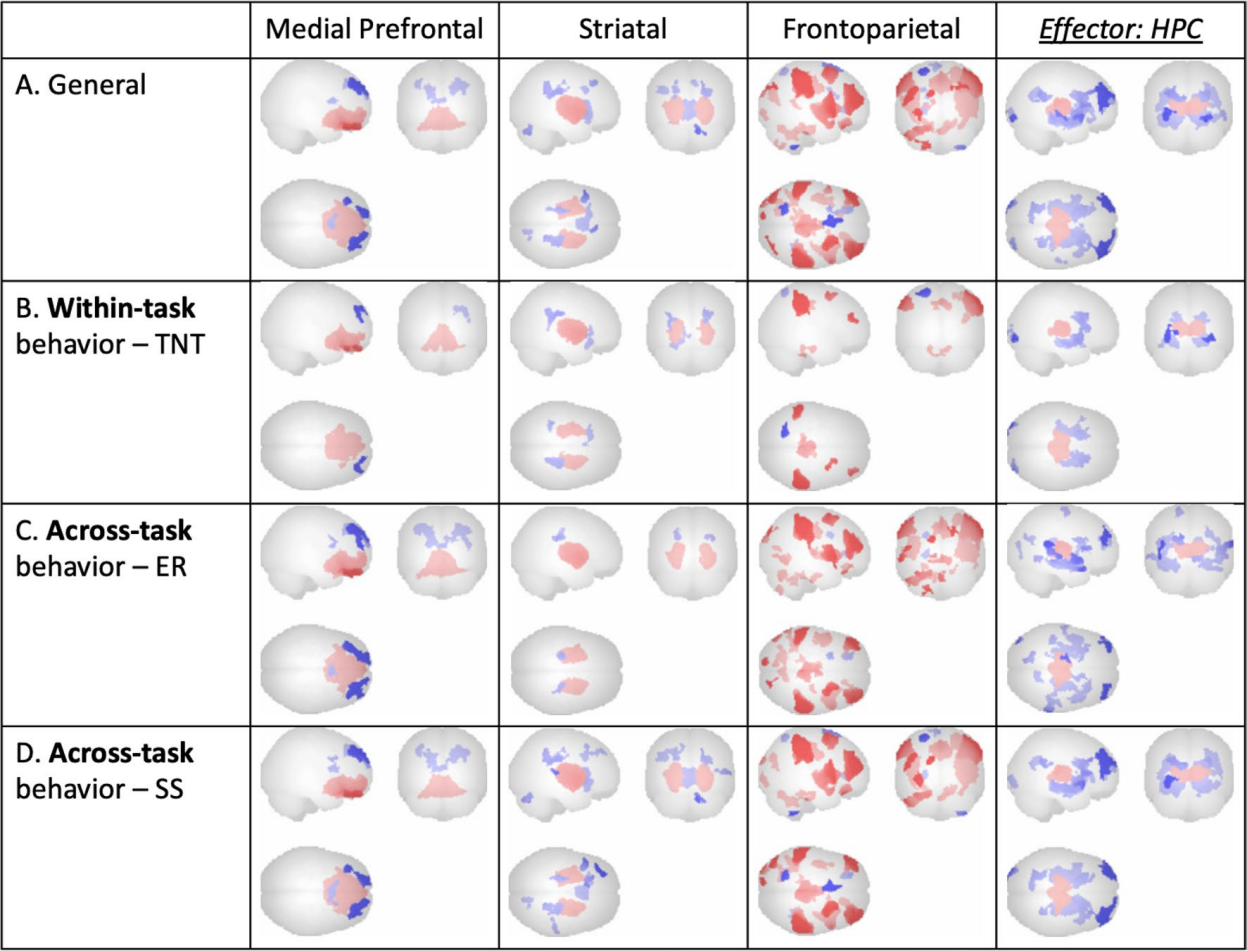
TNT No-Think condition ICNs: Across-task irrespective of behavioral measures (‘General’; **A**) and regressed with within-task (**B**) and across-task (**C**, **D**) behavioral measures, all at voxel threshold p < 0.001, cluster threshold p < 0.05, cluster-size p-FDR corrected, |T(17)| = 3.97 (Gaussian Random Field theory; Worsley et al. [47])

Furthermore, three specific components exhibited negative connectivity of the timeseries in putative key effector regions with the ‘general’ 13 components for each of the three tasks (ER: AMY, TNT: HPC, SS: PMC). These three effector components were selected for further examination using both visual inspection and ROI masks from CONN. The selected components are presented in the last columns of Figs. 3 and 5, and 7.

Subsequently, the components were regressed with behavioral suppression measures from the three tasks (ER: difference between baseline rating and suppression rating, TNT: difference between baseline performance and recall accuracy during the No-Think condition, SS: stop signal reaction time) to identify commonalities across tasks in brain-behavior relationships. These relationships are depicted in Figs. 3b**–**d and 5b**–**d, and 7b**–**d. These final components will be referred to as ‘ICNs’ henceforth.

### Emotion regulation task results

In addition to ICNs ubiquitous across all tasks (a medial prefrontal network, a striatal network, and a frontoparietal top-down executive control network), a right-lateralized executive control network coinciding with downregulation in the ER-task-specific effector ROI (AMY) was observed. Task-based ICNs were found to be related not only within the task’s own behavioral measure (Fig. 3b) but also across the other two tasks’ behavioral measures (Fig. 3c, d). Additionally, within-task ROI peaks in the effector ICN were identified and plotted for individual participant component loadings, as well as for effect size of all participants (Fig. 4a–c). To minimize reverse inference and assess the robustness of our findings, canonical 17-network parcellation mask templates [48] were spatially matched to resultant ICNs, with the general medial prefrontal ICN aligned with Yeo Network 12 (‘*Control A’*), the general striatal ICN aligned with Yeo Network 8 (‘*Salience’*), the general frontoparietal ICN aligned with Yeo Network 1 (‘*Peripheral’*), and the task-specific amygdalar ICN aligned with Yeo Network 10 (‘*Limbic’*) (Fig. 3).

**Fig. 6:**
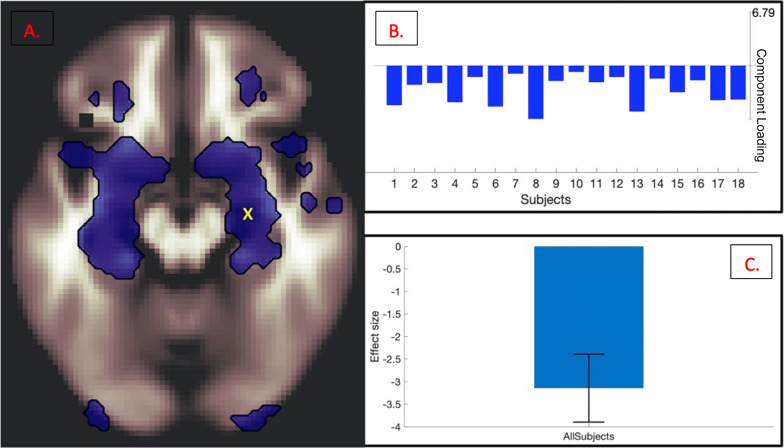
Mean across-participant peak (**A**; marked with a yellow x) of the ER task-specific effector ROI (AMY), plotted for individual participant negative component loading weights (**B**), and for effect size of all participants (**C**), p = 0.000023

These results suggest that self-regulation of emotional reactivity is related to increases in medial prefrontal, striatal, and frontoparietal ICNs, with the addition of a right-lateralized executive control network, which coincides with downregulation in the amygdala.

### Memory suppression task results

In addition to ICNs ubiquitous across all tasks (a medial prefrontal network, a striatal network, and a frontoparietal top-down executive control network), downregulation in the TNT-task-specific ROI (HPC) was observed. Task-based ICNs were found to be related not only within the task’s own behavioral measure (Fig. 5b) but also across the other two tasks’ behavioral measures (Fig. 5c, d). Additionally, within-task ROI peaks in the effector ICN were identified and plotted for individual participant component loadings, as well as for effect size of all participants (Fig. 6a–c). To minimize reverse inference and assess the robustness of our findings, canonical 17-network parcellation mask templates [48] were spatially matched to resultant ICNs, with the general medial prefrontal ICN aligned with Yeo Network 10 (‘*Limbic’*), the general striatal ICN aligned with Yeo Network 7 (‘*Ventral Attention’*), the general frontoparietal ICN aligned with Yeo Network 8 (‘*Salience’*), and the task-specific hippocampal ICN aligned with Yeo Network 11 (‘*Control C’*) (Fig. 5).

**Fig. 7:**
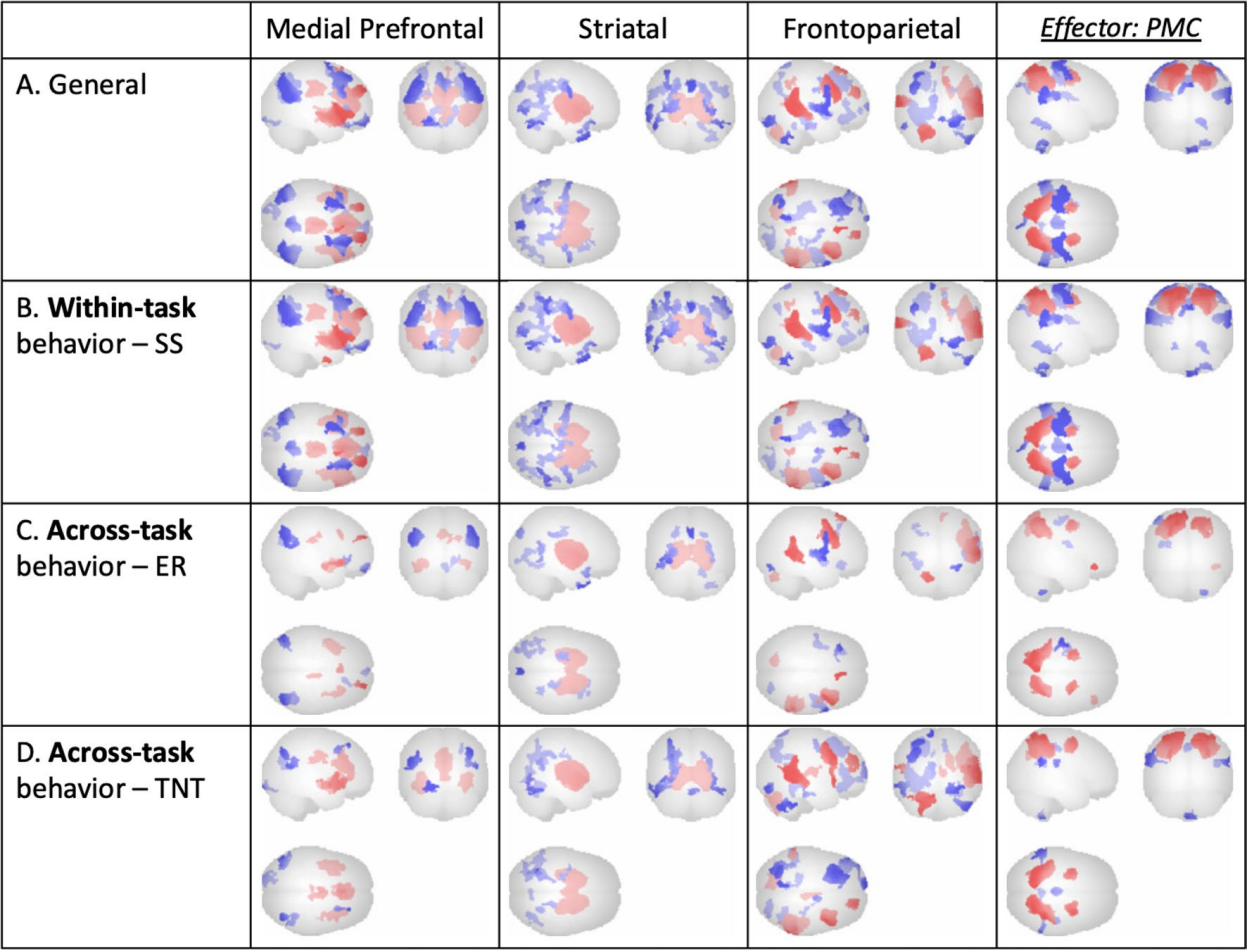
SS attempt (correct + incorrect at a contrast of 0.5 + 0.5) condition ICNs: Across-task irrespective of behavioral measures (‘General’; **A**) and regressed with within-task (**B**) and across-task (**C**, **D**) behavioral measures, all at voxel threshold p < 0.001, cluster threshold p < 0.05, cluster-size p-FDR corrected, F(2, 34) = 8.52 (Gaussian Random Field theory; Worsley et al., [47])

These results suggest that self-regulation of memory retrieval is related to increases in medial prefrontal, striatal, and frontoparietal ICNs, which coincides with downregulation in the hippocampus.

### Motor inhibition task results

**Fig. 8:**
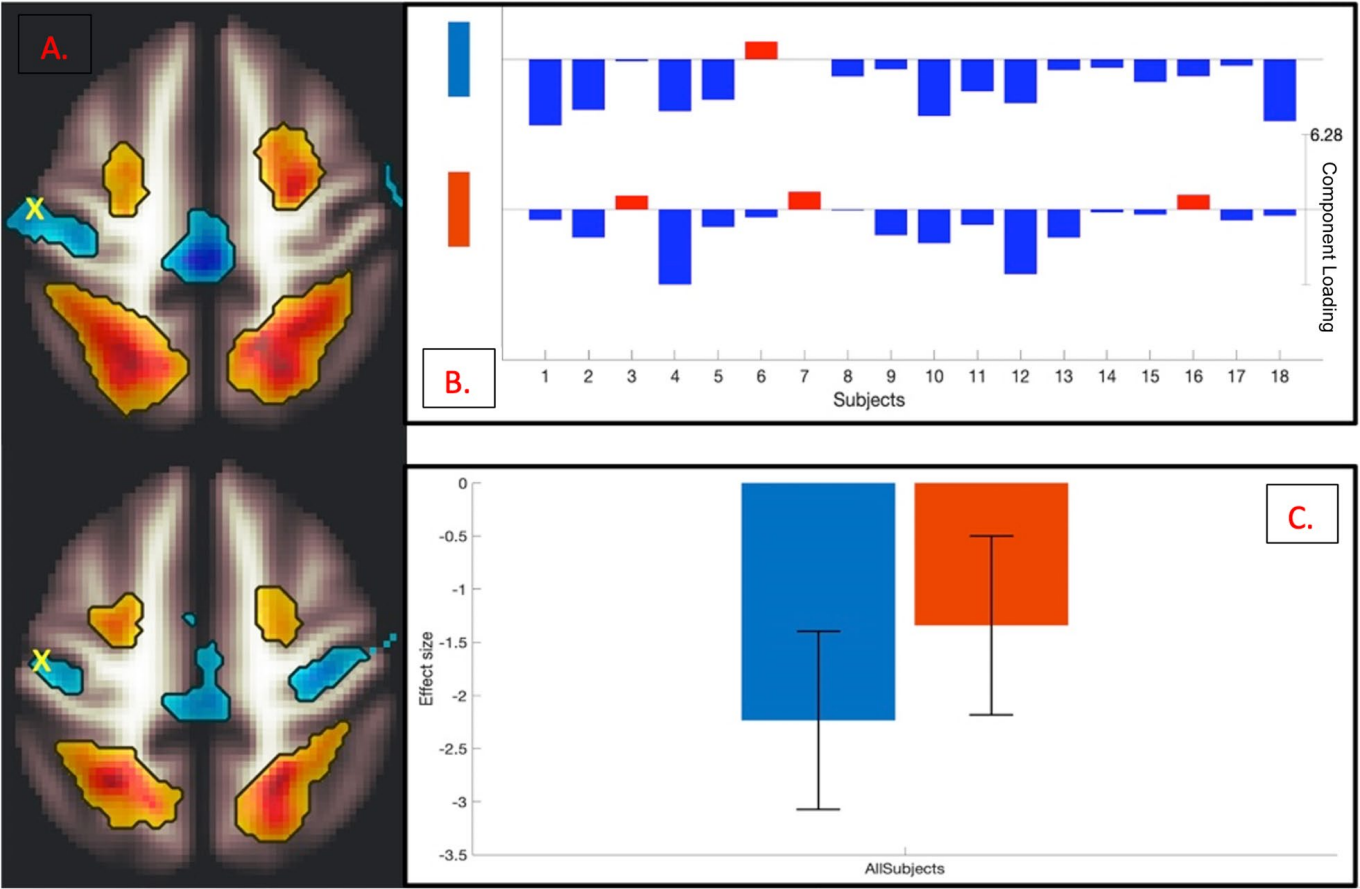
Mean across-participant peak (**A**; marked with a yellow x) of the SS task-specific effector ROI (PMC), plotted for individual participant negative component loading weights (**B**), and for effect size of all participants (**C**), p = 0.000001, at a between-conditions contrast of [0.5, 0.5] for correct (blue) and incorrect (red/orange) responses as ‘attempted accuracy’, p = 0.000236

In addition to ICNs ubiquitous across all tasks (a medial prefrontal network, a striatal network, and a frontoparietal top-down executive control network), an executive control network coinciding with downregulation in the SS-task-specific ROI (PMC) was observed. Task-based ICNs were found to be related not only within the task’s own behavioral measure (Fig. 7b) but also across the other two tasks’ behavioral measures (Fig. 7c, d). Additionally, within-task ROI peaks in the effector ICN were identified and plotted for individual participant component loadings, as well as for effect size of all participants (Fig. 8a–c). To minimize reverse inference and assess the robustness of our findings, canonical 17-network parcellation mask templates [48] were spatially matched to resultant ICNs, with the general medial prefrontal ICN aligned with Yeo Network 8 (‘*Salience’*), the general frontoparietal ICN aligned with Yeo Network 14 (‘*Default D’*), and the task-specific PMC ICN aligned with Yeo Network 6 (‘*Dorsal B’*) (Fig. 7). The general striatal ICN did not map significantly onto any one Yeo network parcellation.

These results suggest that self-regulation of prepotent motor response is related to increases in medial prefrontal, striatal, and frontoparietal ICNs, with the addition of an executive control network, which coincides with downregulation in the primary motor cortex.

### Across-task functional connectivity results

Across-task network commonalities indicated by ICA included a medial prefrontal network, a striatal network, and a frontoparietal top-down executive control network. Additionally, downregulation in each task’s specific ROI was observed. Task-based functional connectivity was found to be related not only to each task’s own behavioral measure but also to the behavioral measures of the other two tasks. The three aforementioned ubiquitous ICNs identified were consistent across-task from the emotion regulation task to both the memory suppression and motor inhibition tasks, from the memory suppression task to both the emotion regulation and motor inhibition tasks, and from the motor inhibition task to both the emotion regulation and memory suppression tasks (Figs. 3, 5 and 7). In both the emotion regulation and memory suppression tasks, the downregulation observed in task-specific ROIs (AMY, HPC) was reciprocally observed across tasks.

## Discussion

The present study aimed to elucidate whether functional connectivity in potentially common underlying executive and cognitive control ICNs could be reliably observed as a result of an increase in task-based behavioral inhibition, as measured by ICA. Results from the ICA analyses of tasks eliciting the regulation of emotional reactivity, memory retrieval, and prepotent motor response indicated that inhibitory control across these domains is related to the ubiquitous recruitment of at least three common neural networks: a medial prefrontal ICN, a striatal ICN, and a frontoparietal ICN. Shared variance in these ICNs was associated with behavioral performance both within- and across-task, relating the identified ICNs to the successful exertion of inhibitory control. While many previous studies have ascertained that inhibitory control requires top-down frontoparietal employment of executive function, our results illustrate that inhibitory control also measurably depends upon the recruitment of additional networks, such as the striatal and medial prefrontal ICNs identified in this study. Furthermore, these results suggest the existence of a global suppression domain underlying multiple facets of inhibitory control.

Downregulation in task-specific ROIs was also observed in analysis results, suggesting the possibility that each common ICN unveiled in this study performs a unique function in the synergic process of downregulating task-specific effector areas. For example, it may be the case that the medial prefrontal network exerts control over reward motivation while the striatal network modulates the updating of the flow of goal-congruent information from subcortical to cortical areas and the frontoparietal executive control network maintains individual goal representations. These areas together potentially facilitate the resultant downregulation observed in task-specific effector areas: the amygdala for emotion regulation (Fig. 4), the hippocampus for memory suppression (Fig. 6), and the primary motor cortex for motor inhibition (Fig. 8).

This study aimed to observe whether functional connectivity in executive and cognitive control ICNs could be reliably observed as the result of an increase in task-based behavioral inhibition and found that inhibitory control across multiple domains is related to the recruitment of at least three common neural networks (medial prefrontal, striatal, and frontoparietal ICNs) that are associated with behavioral performance, suggesting the existence of a global suppression domain underlying multiple facets of inhibitory control. These findings are in line with other recent important works on the topic, such as recent research on inhibitory task overlap illustrating that multiple types of inhibitory control involve similar regions in the right dorsolateral and ventrolateral prefrontal cortex (vlPFC) which dynamically target different brain regions (such as the primary motor cortex or hippocampus) depending on the task at hand, supporting the idea of a single domain-general system underlying inhibitory control [5]. Findings by Wessel et al. in [46] suggested that surprise interrupts cognition via the same fronto-basal ganglia mechanism that interrupts action. Further work by Wessel and Aron [45] proposed that all unexpected events—action mistakes, unexpected action results, or unexpected perceptual events—recruit the same fronto-basal ganglia network for stopping. Adding merit, level-dependent subthalamic nucleus activation has been observed during the recruitment of this network in response to the need for unexpected action or inhibition [40, 46].

### Medial prefrontal network

The mPFC plays an important role in the integration and dissemination of task-salient information. It is implicated in aiding the amygdala in cognitive reappraisal during the regulation of prepotent emotional reactivity [8] and in facilitating both bottom-up and top-down memory inhibition processes such as retrieval-induced forgetting and retrieval suppression [2, 18]. As such, it is to be expected that the medial prefrontal network identified in this study can be reliably observed across both the emotion regulation and memory suppression tasks (likely aiding in facilitating the downregulation observed in the ER-task-specific effector area, the amygdala). However, the role of this ICN in the inhibition of prepotent motor response may seem less intuitive. Although there is no overtly emotional component to the motor inhibition task, a signal must be sent regardless to inhibit a participant’s motor response. Such a signal accompanies a decreased level of dopaminergic activation, allowing a participant to maintain salient information within the context of “goal-directed decision-making and action selection” [41].

### Striatal Network

The striatum is a subcortical structure that plays a critical role in regulating responses to rewarding or aversive stimuli, as it receives input related to these stimuli and controls projections to cortical areas facilitating action selection and initiation. The striatum is often linked to activation in the SS-task-specific effector area (the primary motor cortex) during tasks eliciting motor inhibition [49], likely facilitating the area’s subsequent downregulation observed in this study. Although this response regulation process is often observed in the context of motor response, the striatum can also facilitate ongoing congruence between actions and emotion or memory goals [43], especially in response to conflicting stimuli [31]. “Emotional information can facilitate or interfere with cognitive processes” and the striatum often works in conjunction with the amygdala to aid in response selection and the volitional direction of attention [27], as in the ER task. Furthermore, the striatum has been shown to work synchronously alongside the TNT-task-specific effector area (the hippocampus) to aid in the blocking of spontaneous memory retrieval [25]. These processes are not without neural overlap, as prior research has revealed that the “Think/No-Think and Stop Signal tasks share a common striatal circuitry involved in the cancellation of unwanted thoughts and actions” [28]. Striatal facilitation of these separate but related processes accounts for the reliable observation of this ICN across all three inhibitory task domains.

### Frontoparietal control network

The frontoparietal control network (sometimes referred to as the central executive network or frontoparietal attention network) is “critical for our ability to coordinate behavior in a rapid, accurate, and flexible goal-driven manner” [33]. In this study, the identified frontoparietal ICN is likely holding online the representation of participant suppression goals through interactions with the aforementioned medial prefrontal and striatal ICNs, facilitating the subsequent downregulation observed in all task-specific effector areas. For example, the frontoparietal control network has been shown to activate in conjunction with other networks to exert inhibitory control over hippocampal pathways, and as a result, over memory retrieval [4]. Increases in activation in the frontoparietal control network have been observed across a wide variety of suppression tasks [20], including those requiring volitional control over memory retrieval [11, 32], attentional shifting and stopping of reflexive motor responses [20], and reappraisal of or self-distancing from emotional stimuli [35].

### Future directions and limitations

Numerous opportunities exist for further research on the neural mechanisms underlying different domains of inhibitory control. One valuable future direction would involve exploring the techniques participants employ to inhibit their prepotent reactions. For example, during the ER task in this study, participants were instructed not to close their eyes or look away from the presented images. However, they were not offered additional guidance on potential techniques for suppressing their emotional reactions. If a global suppression domain does indeed exist, as our results suggest, it would be worthwhile to uncover the techniques participants employ to successfully exert global inhibitory control. The identification of these techniques could be a contributory asset to the development of novel interventions for clinical dysfunctions in self-regulation.

However, comparisons between clinical and control samples would be necessary for the present findings to be clinically translational in nature. All participants in the present study reported no history of psychiatric disorders, and it would be worthwhile to compare various domains of inhibitory control in a sample of participants diagnosed with clinical mental health disorders. Another limitation of the present study is an inadequate sample size for analyzing measures of individual differences. Although well-documented gender differences exist in processing salient stimuli [34], analysis of these differences is beyond the scope of this study due to the aforementioned small sample size. Lastly, measures of suppression difficulty and emotional intensity were self-report in nature, and thus subject to the limitations of self-report indices such as participant bias and individual variability in levels of self-awareness.

### Summary and conclusions

Our research provides evidence for the existence of a global suppression domain underlying multiple types of inhibitory control. Data-driven ICA results reliably indicated medial prefrontal, striatal, and frontoparietal ICNs ubiquitously present in three tasks eliciting inhibitory control (emotion regulation, memory suppression, and motor inhibition), presumably facilitating the observed downregulation in task-specific effector regions (the amygdala for emotion regulation, the hippocampus for memory suppression, and the primary motor cortex for motor inhibition). Further research is necessary to fully illuminate the neural underpinnings of inhibitory control and aid in the development of interventions for various clinical mental health disorders.

## Data Availability

The datasets analyzed during the current study are available at https://openneuro.org/.
